# A narrative review of barriers to and promotion strategies for female college students’ sports participation in a cross-cultural context

**DOI:** 10.3389/fpubh.2025.1658796

**Published:** 2025-10-02

**Authors:** Jiajin Zhou, Chuchen Liu

**Affiliations:** ^1^Institute of Physical Education and Health, Yulin Normal University, Yulin, China; ^2^College of Physical Education and Health, Guangxi Normal University, Guilin, China

**Keywords:** female college students, sports participation, barriers, cross-cultural context, narrative review

## Abstract

Female university students face significant barriers to sports participation across diverse cultural contexts, despite its well-documented benefits for physical and mental health. Through a narrative review of 18 studies, this paper synthesizes barriers and identifies multi-level promotion strategies. Findings reveal four interconnected dimensions of obstacles: (a) Physiological barriers, including menstrual health concerns and heightened injury risks due to inadequate skills/knowledge; (b) Psychological barriers, characterized by body image anxiety, low self-efficacy, and trauma from negative past experiences; (c) Sociocultural barriers, manifesting as gendered stereotypes, lack of social support, and religious-cultural norms restricting participation (e.g., modesty requirements); and (d) Environmental barriers, encompassing male-dominated facilities, unsafe/inaccessible spaces, and climate-related discomfort. To address these challenges, the review proposes integrated interventions: (a) Physiological strategies include cycle-adapted exercise plans and injury-prevention education; (b) Psychological strategies focus on trauma-informed cognitive restructuring and graduated achievement systems; (c) Sociocultural strategies involve deconstructing gender biases through media representation and creating faith-sensitive spaces (e.g., women-only facilities with visual isolation); (d) Environmental strategies prioritize gender-responsive spatial redesign (e.g., reserved time slots, repurposed underutilized areas). This promotes female college students’ enthusiasm for sports participation and even encourages them to reconstruct the socio-cultural pressures they face.

## 1 Introduction

Sports participation includes any form of physical activity that involves skeletal muscle movement and requires energy expenditure (1). Sports participation includes many types of exercise, including vigorous and moderate-intensity activities aimed at improving physical health (2).

The positive value of sports participation for women’s health has been verified in numerous empirical studies. For example, sports participation has a certain positive effect on menstrual health, such as more regular menstruation and reduced menstrual discomfort in athletes (3). In addition, in terms of mental health, female college students who regularly participate in tennis training not only improve their self-esteem but also significantly reduce depressive symptoms (4). Physical activity can also effectively improve physical symptoms, anxiety, sleep disorders, and impaired social functioning in female college students who are not physical education majors (5). Subsequent studies have also pointed out that sports participation can greatly improve female college students’ speed, agility, endurance, abdominal strength, and leg strength (6). These findings clearly indicate that physical activity not only helps improve physical fitness, but is also an important tool for college students’ psychological adjustment.

According to the results of regular tests conducted by the World Health Organization (WHO), adolescent students, mainly college students (17–23 years old), have low levels of physical activity (7). For example, 78% of adolescent boys whose physical activity mainly consists of sports participation do not meet the WHO’s recommended level of physical activity (7). Adolescent girls in the college student group have even lower levels of physical activity, with only 15% of adolescent girls meeting the requirements (7). Similarly, a large number of scientific studies have confirmed the findings of the WHO investigation. Compared with male students, female college students still participate in sports less frequently and in a more limited range of activities, mostly focusing on low-intensity exercises such as aerobics, yoga, and running (8). Specifically, in a study conducted at Kaluga State University, only 21 of 148 female college students rejected any form of sports participation (9). This shows that although most students are aware of the importance of participation, there are still issues with frequency and consistency (9).

A study conducted at Yarmouk University of Jordan found that approximately 56.7% of female college students said they did not participate in any form of sports, and only 24.3% participated in sports activities on a regular basis (10). Other studies have found similar results. For example, Memon et al. (11) surveyed female college students at the Peoples University of Medical & Health Sciences for Women and found that only 28 (22%) participants said they regularly participated in sports. Another study of 60 female college students at University Technology Malaysia also found that only 31.7% of female college students considered sports participation to be important (10). The data from Leslie-Walke et al. (12) is even more serious. They surveyed female college students from West Africa studying in the UK on their sports participation and found that 70% of female college students did not participate in any sports activities during their college years.

It should be noted that the WHO also pointed out that this phenomenon has continued for 12 years (2010–2022) (7). If the level of physical activity among adolescents, such as college students, is not effectively improved, more adults (reaching or even exceeding 35%) will be severely physically inactive (7). For example, Leslie-Walke (12) found in a follow-up survey that 58% of female college students still did not participate in any sports activities after graduation.

To this end, the academic community has begun to study the specific circumstances facing female college students’ sports participation from physiological, psychological, cultural, and environmental perspectives (9, 10, 12). However, it should be noted that current research lacks a sorting and analysis of cross-cultural differences and inclusive intervention measures. Most existing studies are based on the culture of their own country or the experience of developed countries, and lack discussion of cross-cultural differences and inclusive interventions. To this end, we aim to leverage the capacity of narrative reviews (narrative review) to flexibly collate findings from interdisciplinary and cross-cultural research, enhancing the readability of literature synthesis and making it accessible to non-specialist audiences (13). We explore the potential barriers that female university students may encounter when engaging in physical activities, and how future efforts could promote their sustained participation. In doing so, we attempt to offer practical insights and theoretical support for improving physical activity participation levels among female university students in the future.

## 2 Methods

In this review, we will focus on the barriers faced by female college students in sports participation. Therefore, we used a narrative review method for analysis. The purpose of this type of review is to provide a comprehensive and easy-to-understand overview of research progress on a specific issue to summarize its theoretical basis or practical experience (14). The narrative method includes summarizing, comparing, explaining, and describing the barriers faced by female college students in sports participation, and analyzing the underlying challenges.

We began our literature search in the Web of Science database in June 2025. Based on the primary focus and research questions of the study investigators, we searched relevant databases or publishers, such as BSCO-ERIC, Scopus, MDPI, Taylor & Francis Online, and Sage Journals. In order to ensure the reliability of the content of this study, we firstly screened from the Web of Science Core Collection, which has a rigorous peer-review system and has high research quality and originality. The search strategy is as follows: sport participation (Topic) and female college students (Topic) and 1996–2025 (Year Published) and Article OR Review (Document Type) and English (Language) = 146. The retrieved literature was sorted, and the exclusion criteria were based on reading the article titles and abstracts, removing literature that was clearly unrelated to the main focus of this study, leaving 85 articles. Subsequently, the full text of the documents was read and screened through peer discussion and back-to-back analysis. First, literature that clearly defined female college students (or including female college students) as the main research subject of sports participation was screened (*n* = 68). Second, literature that explicitly mentioned the factors promoting or hindering sports participation among female college students (or including female college students) was further included in this study (*n* = 66). Finally, to analyze and discuss the various factors influencing the sports participation of female college students across different cultures, some research with a high degree of repetition, or that had been conducted repeatedly in certain countries, was excluded. The factors promoting or hindering sports participation among female college students (or including female college students) mentioned in all the literature were summarized (*n* = 18, the specific content is shown in Table 1).

**Table 1:** Summary of factors promoting and hindering sports participation among female college students.

	Citation information	Research subjects (nationality)	Promotion	Barriers
1	Motevalli et al. (26)	4,508 participants (mean age: 24.9 years; 65.9% female) from 52 Austrian colleges/universities. (Austrian)	Maintaining physical health (12.9%); feeling good (11.3%); refreshing the mind (10.0%); maintaining body shape (5.1%); enjoying sports engagement (6.0%)	N/A
2	Brunton and Quinton (30)	6 focus interview groups, each consisting of 6 to 9 female college students who do not often participate in sports. (England)	To feel welcome to be part of the team or club; to be able to make or spend time with friends; building confidence; bespoke communications; More opportunities; better pricing; distance to travel	N/A
3	Carson et al. (25)	3,277 female-identifying students at 12 colleges and universities. (Greek)	High-intensity competitive sports may be due to high demands for performance, intense training, psychological stress, and possible inadequate energy intake, all of which combine to increase the risk of menstrual disorders.	Proper physical activity can reduce the chances of menstrual disorders in women.
4	Leslie-Walker et al. (12)	168 participants with an average age of 34 years (20–46 years) complimented by two focus group interviews involving 15 participants. The female students were required to be born in West Africa and undertaking an undergraduate program at the England university. (West Africa)	Personal intrinsic motivation; past sports experience (in West Africa); improvement of health awareness	Cultural differences; insufficient publicity and promotion; time arrangement conflict; lack of companionship; cost concerns; lack of sports equipment
5	Yang et al. (28)	483 non-physical education majors undergraduate students (mean age = 19.71 ± 1.31 years, 56.1% female) from five randomly selected universities in Wuhan.(China)	Conscientiousness; extraversion	Neuroticism; agreeableness showed no significant association; exercise motivation
6	Ma et al. (27)	500 undergraduates (256 males, 244 females) from five universities in Shanghai. (China)	N/A	Female undergraduates showed the lowest PA levels in masculine sports (*p* = 0.002). Among female participants, gender roles significantly affected PA and involvement, with androgynous individuals performing best, especially in masculine sports (PA: *p* < 0.001; involvement: *p* < 0.001). Feminine and undifferentiated roles were associated with lower scores in value cognition (*p* < 0.001) and autonomy (*p* < 0.001).
7	Fakehy et al. (17)	645 female students from universities of Saudi Arabia. (Saudi Arabia)	Physical fitness, social experience and formal competition have a positive effect on physiological experience which in turn has a positive effect on the attitude of female students towards sports.	N/A
8	Kamal et al. (10)	60 female students were from the Faculty of Education, University Technology MARA. (Malaysia)	Perceived benefits of physical activity; interest in physical activity; preference for outdoor activities.	Time constraints; poor personal status; insufficient objective conditions; sociocultural norms.
9	Memon et al. (11)	403 students from the Peoples University. (Pakistan)	Intrinsic motivation; extrinsic motivation; health benefits.	Academic pressure: high academic demands and pressure (90.6%); gender differences; lack of exercise habits (22%) inadequate field facilities; fear of sports injuries; technical and environmental factors.
10	Turkmen, 2018 (23)	412 university students attending to different faculties in Bartin University in Turkey. (Turkey)	Societal perceptions change; religious attitudes are supportive; sporting achievements motivate.	Traditional gender roles; geographical and cultural differences; perceived religious-related limitations; limited choice of sports.
11	Lin and Liu (31)	1,200 female university students. (China)	Daily exercise time; regular exercise habits; self-health awareness.	Age; daily internet time; the overall status quo: at present, female college students generally lack of leisure activities and sleep, and the ratio of regular fitness habits is low, and the number and average daily internet access time are generally too high, these factors together lead to the worst performance of sports participation behaviors among female college students.
12	Agbabiaka et al. (29)	291 respondents from Bayero University. (Nigerian)	Getting exercise (SPI = 4.41), keep physically fit (SPI = 4.39), educational/research (SPI = 4.38), improving physical, and emotional health (SPI = 4.29), developing skill/ability (SPI = 4.08), boredom (SPI = 3.99), and minimize stress (SPI = 3.77)	Restricted access to sports facilities; cultural and social constraints; cultural and social constraints; time and family responsibilities; lack of awareness and guidance.
13	Mirsafian et al. (22)	This study aims to explore the attitude of Iranian female university students toward sport by survey method (*N* = 1,120) and qualitative interviews (*N* = 50). (Iranian)	N/A	Societal level; level of religious norms; facility level; individual level.
14	Kanaan et al. (21)	90 Jordanian female university students. (Jordan)	Family support; Islam itself encourages physical activity, but only in a gender-segregated environment, which provides a cultural rationale for promoting women’s sports under the right conditions.	Insufficient time; lack of sports facilities and clubs; negative attitudes towards sports; religious and cultural factors.
15	Park et al. (8)	494 female undergraduate students attending Kyung Hee University in South Korea. (Korean)	Facilitating factors are mainly based on the fulfilment of psychological needs and personal cognitive factors. Examples include: competence, autonomy and relatedness.	Subjective norm—i.e. the individual’s perceived expectations of their behavior from significant others
16	Kv et al. (6)	40 undergraduate women. (India)	Flexibility; economy; demonstrable health benefits	Individual health problems; personal commitments; fixed nature of time; place of sports participation
17	Samara et al. (24)	2000 female university students. (China)	N/A	Individual physiological factors; participation and support from those around them; site and natural environment factors; lack of female sports facilities and exercise opportunities.
18	Homai et al. (3)	360 girl students in 18–28 years old majoring in medicine and non-medicine at the universities of Tabriz. (Iran)	For the average female university student: improvement of dysmenorrhea; reduction of analgesic use.	In athletes (who are under more athletic stress) intense or early physical training may lead to menstrual disorders or delayed menarche by lowering body fat percentage (e.g., below 17 per cent), increasing psychological stress, or negative energy balance.

In this narrative review study, the analytical logic of thematic synthesis was used to summarize and synthesize existing research findings. Thematic synthesis involves three key stages—line-by-line coding, development of descriptive themes, and generation of analytical themes—which together ensure fidelity to original findings while allowing deeper theoretical insights (15). Specifically, this study sorted and analyzed the influencing factors and obstacles to female college students’ sports participation in existing research results, and summarized the relevant content into four dimensions: “physiological,” “psychological,” “sociocultural,” and “environmental.”

## 3 Analysis of barriers to sports participation among female college students

### 3.1 Barriers related to physiological factors

Female college students face many physiological barriers to sports participation. These barriers stem not only from individual physiological characteristics, but also from interactions with the sociocultural environment, which significantly inhibit their willingness and behavior to participate. Among these, physiological barriers mainly include menstruation-related issues and the risk of sports injuries, which can be systematically analyzed from the perspective of the social-ecological model. Li et al. (16) used the social-ecological model to explore the internal barriers to female college students’ participation in sports activities, emphasizing that key issues at the physiological level include the influence of personal external image and physical condition.

Specifically, female college students often avoid participating in physical activities or sports due to weakness and hormonal imbalances caused by menstruation (Factor Loading, FL = 0.800), a phenomenon supported by empirical research in the literature (16). For example, a survey of female college students aged 18–22 in China showed that physiological factors such as menstruation are major barriers, causing many students to voluntarily give up exercise during their menstrual periods, resulting in intermittent sports participation (16). This intermittent participation not only reduces overall activity frequency but may also trigger a vicious cycle: menstrual discomfort exacerbates negative emotions such as fatigue and pain, further weakening motivation to exercise. Romanova and Emelyanova (9) further expanded on this, finding that some female university students who engage in regular exercise experience delayed menarche, suggesting a complex relationship between exercise and physiological development. Their research indicates that delayed menstruation may stem from the disruption of the endocrine system caused by high-intensity exercise, but this “protective” effect only applies to specific groups. Most female college students avoid exercise due to menstrual discomfort, thereby inhibiting their willingness to participate (9).

In addition, female college students’ physical fitness, lack of knowledge and skills in sports significantly increase the risk of sports injuries, which constitutes another core physiological barrier. Sports injuries not only cause physical pain, but also trigger psychological fear, forming a “pain-avoidance” cycle that further inhibits sports participation (17). A study from Pakistan provides compelling evidence: a cross-sectional survey of 403 female college students revealed an injury incidence rate as high as 31.51%, with the most common types being cramps (46.5%) and sprains (45.7%). The primary causes included improper exercise techniques and insufficient warm-up (11). These data highlight the severity of skill deficiencies—for example, ankle sprains in sports often result from technical errors such as improper landing posture, while cramps are associated with dehydration or excessive fatigue (18). This risk of injury is particularly prominent in groups that lack regular exercise, as they have lower physical adaptability and are prone to injury during sudden activities (19). Chinese research in the knowledge base echoes this point: individual physiological factors (such as “menstruation”) are indirectly related to sports injuries, as physiological weakness during menstruation may reduce motor coordination and increase the probability of mistakes (16). More importantly, the psychological consequences of sports injuries cannot be ignored: a study based on the Athlete Fear Avoidance Questionnaire (AFAQ) found that female college students showed higher fear avoidance scores after injury, which led them to “convince” themselves to refuse to participate in sports activities to avoid pain (20). This psychological mechanism may form a long-term barrier to participation.

### 3.2 Barriers related to psychological factor

Female college students’ self-identity and body image anxiety constitute fundamental barriers to their daily sports participation. The core contradiction of this barrier lies in the conflict between the socially and culturally constructed conservative culture of women and the design of sportswear (16). For example, in a Chinese study, some female college students refused to exercise because they were unwilling to expose their bodies during sports and physical activities (FL = 0.661) (16).

At the behavioral motivation level, the absence of psychological resilience and cognitive biases create persistent barriers. Female college students are more likely than males to discontinue exercise programs (4). The root causes lie in two main aspects: first, female college students typically have lower self-efficacy (e.g., “unable to perform complex movements”); Second, they have less perseverance than male college students (4). This trait is closely related to misperceptions—most female college students view physical activity as an “extreme sport” that is difficult to master, and therefore reject participation in physical activity (10).

This misconception also leads to a lack of psychological preparation for sports participation. A cross-sectional survey of female West African university students studying in the UK found that Joy said, “I did not do much sport back home either, I chose lessons after school that helped me to learn how to sew clothes. Sport is not really my thing, but since I’m training to be a nurse, I understand why it’s important for your health… I would probably like to try something like badminton” (12). This indicates that many female university students fail to recognize the importance of physical activity for their studies and daily lives.

In addition, the negative reinforcement of emotional memories during sports participation further constitutes a deep psychological mechanism that causes female college students to reject sports participation. Unpleasant past experiences with sports also inhibit female college students’ willingness to participate in sports. For example, mistakes made in physical education classes due to personal reasons can cause their sensitive emotions to perceive negative emotions such as “embarrassment” and “shame.” When such obstacles are compounded by academic pressure and social anxiety, it creates psychological internal friction, making sports participation a symbol of emotional burden (21), causing many female college students to avoid participating in group sports.

### 3.3 Barriers related to social factor

Sociocultural stereotypes regarding women’s participation in sports continue to pose a deep-rooted obstacle. Cross-cultural studies show that some groups still view women’s participation in strength-based or competitive sports as “inelegant” and “inappropriate,” and this prejudice significantly dampens female college students’ enthusiasm for participation (9). In the Chinese university environment, parents and teachers generally support female students’ participation in sports less than male students, and the curriculum system emphasizes theoretical teaching and weakens practical training, further reinforcing social structural barriers (16). These gender role expectations are continuously transmitted through social construction.

The personality characteristics of women make female college students more directly and profoundly affected by interpersonal networks. For example, they may choose whether to participate in sports based on the sports participation of those around them and the support they receive from others. Specifically, existing research points out that female college students’ sports decisions are highly dependent on social support systems, and their willingness to participate is strongly correlated with peer participation (FL = 0.812), family member attitudes (FL = 0.746), and the values of their social circles regarding sports (FL = 0.821) (16). When environmental changes disrupt existing social structures, participation habits often cease—as in the case of West African students in British universities, where Sade candidly stated: I exercise less now than when I lived back home; I do not walk or run as much because I drive” (12). This disruption is particularly pronounced among cross-cultural study groups, with 45% of respondents in a Jordanian study abandoning exercise due to the loss of their original exercise partners (21).

On the other hand, social sports and the atmosphere of sports activities also affect female college students’ attitudes toward sports participation. In general, female college students commonly express their perception of male dominance in the field of sports (FL = 0.802) and their concern about possible harassment in public places (FL = 0.554) (16).

In different sociocultural contexts, religious beliefs and local sociocultural values shape the way they construct and treat life, influencing female college students’ attitudes and willingness to participate in sports, reflecting more profound cultural constraints (22). Taking Islamic culture as an example, because Iran is a country with “generally strict” management of women’s physical exercise among Islamic countries, the degree of obstruction to female college students’ sports participation is not high, but there is still a certain impact (3). For example, Kanaan et al. (21) conducted nine interviews with 90 female college students in Jordan through semi-structured focus group interviews and found that the Muslim culture of the local society is an important factor preventing female college students from participating in sports. In the interviews, they found that about 45% of female college students had a negative attitude toward sports participation, such as “I do not like sport because the physical education teachers in school did not encourage us to love sport, in addition the school facilities at that time were not encouraging” and “I did not practise physical activity in the early stages, teachers never taught us the benefits of sport, PE lessons were not implemented” (21). This phenomenon is related to the influence of Jordanian traditional culture on women. The study mentioned specific views of female university students, such as “doing physical activities in an open area clash with my religious beliefs” and “I am unable to participate in physical activity if I can be seen by men” (21).

Female college students in different countries have similar views. For example, female college students at the University Technology Malaysia said that their social, religious, and cultural norms restricted them from participating in sports such as swimming (10). This is because in sports such as swimming, they must wear swimsuits that expose large areas of skin, which makes them feel uncomfortable, especially in the presence of men (10).

It is worth noting that the influence of religion is dialectical in nature. For example, Turkmen (23) conducted a questionnaire survey on religious beliefs and sports participation among 412 female college students at a Turkish university and found a weak positive correlation between the degree of religious belief and sports participation. This suggests that moderate religious activities may promote physical practice through collective rituals (23). This complexity prompts us to move beyond the simplistic notion that “religion is an obstacle” and instead explore the promotional elements within religious culture.

### 3.4 Barriers related to environment factor

Environmental barriers to female college students’ sports participation stem from structural imbalances in the environment. There are significant gender differences in the allocation of sports resources in colleges and universities, with most sports facilities giving priority to male-dominated competitions and training, resulting in a systematic reduction in women’s access to resources. Generally speaking, except for a few schools with more resources, most female college students face limited access to sports facilities, and this imbalance is further manifested in physical space constraints. For example, Li et al. (16) found that many female college students reported facing issues such as outdated and unsanitary facilities (FL = 0.875), outdated and remote sports facilities (FL = 0.866), overcrowded sports facilities (FL = 0.861), inconvenient transportation to exercise venues (FL = 0.764), and safety hazards in facilities (FL = 0.763).

In addition, the natural environment also affects female college students’ willingness to participate in sports, such as direct sunlight in summer, rainy or windy weather, and cold weather in winter (16). This point was also highlighted in Kamal et al.’s (10) study, which found that female college students worry about sunburn or skin darkening from direct sunlight, and the image anxiety caused by UV exposure leads women to associate physical activities with “skin darkening = diminished attractiveness.”

Furthermore, some universities lack suitable venues and facilities, depriving students of the conditions necessary for exercise and ultimately causing them to give up trying. For example, Mary stated, “I loved doing track when at school, it does not seem as popular over here. I have never seen a track and field facility, but that would be good as it would bring back good memories” (12). Similarly, some universities lack promotion and information about sports equipment, sports field locations, and other facilities, forcing female college students to invest additional time and resources to participate in physical activities. One interviewee, Margaret, stated, “Well…I do not think there is much advertising. I saw people advertising sports at the fresher’s fair (university orientation), but I have not seen any advertising since. Some of the information said sports hall, but I have no idea where the sports hall is.” (12) This disconnects in spatial awareness forces women to spend extra money exploring venues, ultimately undermining their motivation to participate in sports.

## 4 Countermeasures for barriers to sports participation among female college students

The results of the literature review revealed that depending on the situational nature of parental involvement in sport parenting, it was primarily on the field of play, on the way home, and in private spaces. These studies found that positive and negative sport parenting in various scenarios can play a significant role in a child’s motor development and personal growth. Therefore, parents need to control their behaviors and emotions in these scenarios in order to achieve effective sport parenting.

### 4.1 Countermeasures for physiological barriers

To address physiological barriers to sports participation among female college students, multi-level strategies based on the social ecological model need to be designed, ranging from individual education to environmental optimization, to alleviate barriers and increase participation rates.

First, for menstruation-related barriers, the core strategy is to strengthen science education and information dissemination to correct misconceptions and promote adaptive exercise programs. For example, incorporating a “Women’s Physiology and Exercise” module into university physical education courses, covering the benefits of moderate exercise (such as improving mood and reducing fatigue) and safety guidelines for exercise intensity (24). Existing research suggests that such education should incorporate cultural sensitivity—for example, using social media to disseminate scientific information and emphasizing the positive impact of exercise on hormonal balance (16). Additionally, developing personalized exercise plans is crucial: Romanova and Emelyanova (9) advocate adjusting activity types based on the menstrual cycle (e.g., recommending low-intensity yoga during menstruation), which can reduce feelings of weakness and maintain participation continuity. Furthermore, schools should mandate that sports facilities provide menstruation-friendly resources, such as hygiene products and rest areas, to minimize barriers related to women’s physiology (3). Another important consideration is concerns related to physiology and health. For example, the relationship between participation in school teams, dance or club activities, and menstruation or other physiological issues can influence some female students’ decisions to participate. Schools should provide health education and support services, as well as opportunities for participation (25).

Secondly, regarding the risk of sports injuries, the focus should be on skill training and preventive education to enhance physical fitness and reduce technical errors. Saragiotto et al. (19) emphasize that structured training can significantly reduce the likelihood of sports injuries, particularly common issues such as sprains and cramps. Specifically, university curricula should incorporate “fundamentals of sports medicine” content, teaching proper techniques (such as landing cushioning methods) and warm-up procedures (18). Memon et al. (11) also confirmed this view, finding that a significant proportion of sports injuries stem from technical errors, and thus recommended embedding real-time coaching feedback into sports activities to correct movements. In addition, psychological intervention is also indispensable—based on AFAQ research, cognitive behavioral therapy can help female college students rebuild their confidence in sports (20).

Thus, through the comprehensive use of these methods, physiological barriers can be effectively alleviated and sports participation among female college students can be promoted.

### 4.2 Countermeasures for psychological barriers

Addressing psychological barriers requires a multi-faceted, coordinated intervention strategy. The primary objective is to provide maximum psychological support for female college students. For example, in conservative cultural regions (such as Jordan), providing women-only facilities and flexible dress codes can reduce conflicts between physical activity participation and women’s religious and cultural norms (21).

To address the lack of confidence in their abilities, a step-by-step achievement system needs to be established. For example, by setting small weekly goals for sports participation (such as “complete 3 sets of 10 min of rope jumping”), female college students can gradually build their willingness to participate in sports and their psychological resilience. Alternatively, group sports can be used to form mutual support groups of 5–7 people to increase peer support for sports participation, thereby reducing feelings of frustration and helplessness during the process of participating in sports. Specifically, universities can lower the psychological threshold for initial participation by establishing “women-only sports communities” or dividing interest groups by discipline (such as a medical school women’s sports club). The strategy of ‘group support + association’ here is consistent with the large sample study on the determinants of university students’ participation in amateur and club sports, and emphasises the importance of associations and mutual support networks in sustaining female university students’ participation in sport (26).

At the same time, the theory of staged behavioral intervention can be introduced to emphasize the benefits of sports in alleviating academic stress and improving concentration during the “hesitation stage.” During the “action stage,” a peer incentive system (such as a sports check-in point system) can be used to strengthen the sense of belonging (4).

To improve female college students’ perception of sports participation, universities should highlight “light exercise” scenarios (such as campus walking and dance classes) in their promotional materials, incorporate sports awareness training into orientation courses for new students, and invite female sports experts to give health lectures specifically for them to raise female college students’ awareness of sports participation through personal experience (27).

In addition, traumatic memories associated with female college students’ sports participation need to be rebuilt through cognitive behavior. First, sports courses should be used to promote the re-experience of female college students’ sports participation (recreating the scene in a safe environment). Second, gradual guidance and teaching should be used to establish positive emotional markers for them (covering negative memories with positive experiences). Then, actively use the teaching process to reconstruct the meaning of memories of failure (defining “failure” as a learning process). And share stories with peers who have had similar experiences to reduce the harm of negative emotions. In addition, individual personality traits influence exercise behavior through self-efficacy and exercise motivation. Therefore, when designing cognitive behavioral interventions and setting stage-based goals, attention should be paid to both enhancing self-efficacy and stimulating intrinsic motivation (28). This will help female college students overcome traumatic memories from sports participation and develop a habit of participating in sports.

### 4.3 Countermeasures for social barriers

To eliminate social barriers and promote sports participation among female college students, it is first necessary to improve gender bias in sports and reconstruct the narrative of sports culture. First, with the help of university publicity departments and sports organizations, documentaries showcasing diverse images of women in sports (such as strength training, soccer, and other traditionally “male” sports) should be produced and released on campus media platforms. These documentaries should subtly emphasize female college students’ strategic thinking and team leadership in sports competitions, weaken the single aesthetic standard of “elegance,” and provide female college students with good psychological preparation for participating in sports activities.

Subsequently, a resilient social support network for female college students’ sports participation should be built to strengthen the sustainability of sports participation. For example, cross-cultural sports communities can be established in international schools to create “sports culture exchange groups” for female college students from different countries, where local students can lead them in experiencing their country’s sports activities and invite them to share the characteristics of their home countries’ sports (such as traditional West African sports dances or traditional Asian sports and cultural activities). This is because large-scale research and practice show that community participation, such as through clubs, interest groups and cross-cultural associations, can significantly increase the likelihood of college students continuing to play sports. Therefore, colleges and universities should support and regulate these communities (26).

In addition, when female college students generally perceive the male-dominated nature of sports and are concerned about possible harassment in public places, schools and society should work together to create a safe, inclusive, and supportive sports environment. For example, female security guards should patrol around sports venues at night, and emergency call devices should be installed to enhance the sense of security for female college students participating in sports activities after school.

In different societies and cultures, religious beliefs and local social and cultural values impose profound normative constraints on behavior and cognition (22). It is necessary to respect and understand religious beliefs and values in different cultural contexts, while exploring ways to encourage female college students to participate in sports activities while respecting these beliefs. For example, we can try to promote female college students’ participation in sports through policy support and strengthened publicity. Specifically, the government and social organizations should formulate and implement relevant policies to encourage and support female college students’ participation in sports activities. This may include policies on financial support, scholarships, and the construction and maintenance of sports facilities. Through these measures, we can create more opportunities and conditions for female college students to participate more freely in sports activities and enjoy the fun and benefits of exercise. At the policy and advocacy levels, it is important to note that the three elements of ‘accessibility of facilities’, ‘cultural respect’ and ‘health services’ should be integrated, rather than relying solely on cultural persuasion. This reduces participation costs through institutional measures (29).

### 4.4 Countermeasures for environment barriers

In order to solve the dilemma of the environment on female college students’ sports participation, it is necessary to build a gender-responsive environmental support system and rebuild the space resource allocation mechanism for female college students’ sports participation.

First, facility accessibility and usage arrangements are important environmental factors influencing female students’ participation—institutionalized arrangements for facility provision, time allocation, and privacy protection can significantly increase participation rates (especially in university environments where facilities are insufficient or usage conflicts are severe) (29). “Time–space” gender zoning management can be implemented, reserving 50% of indoor venue usage rights for female students during the prime time after school (16:00–19:00). The focus should be on renovating existing basic sports facilities and incorporating privacy protection in changing rooms into safety audit standards.

In terms of the natural environment, consideration can be given to installing low-cost semi-open dome designs and retractable awnings in open-air venues to improve existing sports venues. Second, culturally appropriate sports venues should be designed to mitigate the negative impact of social culture on female college students’ sports participation. For example, in religiously sensitive areas, adopt a “visual separation + independent access” model to build women’s sports centres. This could involve using frosted glass to separate male and female activity areas, ensuring compliance with religious requirements without compromising sports space (23, 24).

Finally, idle spaces should be repurposed. Low-utilization areas such as building corridors and mezzanines can be converted into micro-fitness stations equipped with yoga mats, jump ropes, and other lightweight equipment (16). At night, the underground garage of the gymnasium can be opened simultaneously, with anti-slip flooring laid to create a roller-skating area, addressing the “no place to move” dilemma (29).

In summary, the elimination of environmental barriers must transcend the simple logic of increasing facilities and shift toward gender-conscious spatial production. When sports venues become safe, visible, and culturally appropriate spaces of empowerment, female college students can truly break free from the paradox of “wanting to move but having nowhere to move” and achieve the reconstruction of bodily autonomy. At the same time, the integrated intervention pathway of teaching, community and venue has been shown in several studies to contribute to the development of a sustainable ecology of women’s sport participation (26, 27).

## 5 Discussion

The value of sports participation as an effective way to promote women’s physical and mental health has been fully verified in cross-cultural studies. From a physiological perspective, regular physical activity can significantly improve female college students’ menstrual health indicators, enhance physical functions (such as speed, endurance, and core strength), and alleviate somatization symptoms. From a psychological perspective, sports participation has been proven to be an important intervention to enhance self-esteem, reduce the risk of depression, and improve social functioning (4, 5). However, there is a clear disconnect between this idealized health function and female college students’ sports participation behavior. The sports participation rate of a large number of female college students is much lower than that of males, and it is characterized by “low intensity” (such as yoga and jogging) and “intermittent” participation (8). This situation reveals that sports participation is not simply a matter of individual choice, but a systemic issue involving the interaction and intertwining of multiple dimensions.

Specifically, first, physiological barriers may be reinforced by structural forces in the sociocultural context: the physical discomfort caused by the menstrual cycle could be alleviated through scientific exercise, but the social gaze that women should reduce physical activity and remain quiet suppresses exercise habits (3, 25). The risk of injury due to insufficient basic exercise knowledge exposes the lack of skill training in physical education for female college students (24).

Second, psychological barriers are rooted in the current social and cultural norms: the oppression and control of women’s attire in social culture leads female college students to associate athletic clothing with “exposure and shame.” (22) A more profound impact stems from negative emotional memories—mistakes made in physical education classes are internalized as “socially stigmatizing events,” triggering avoidance behaviors to protect self-esteem. Specifically, normative aesthetics and body ideals create body-image anxieties that can paradoxically both push and pull women toward exercise—some female students engage in exercise to conform to slenderness or whiteness ideals, while others withdraw from public activity to avoid perceived exposure or stigma. In other words, social norms do not only prohibit or permit behavior directly; they act through internalized self-evaluations and embodied anxieties that mediate whether sport is experienced as self-care or as another site of surveillance (10, 22).

Third, the influence of social culture is multifaceted: Islamic regulations on women’s bodies establish exercise taboos (e.g., “no men in public spaces”), but different religious cultures may also promote physical practices through collective rituals (23). Therefore, religious culture has a dual nature in the process of female college students’ participation in sports: while religious dress codes or festival-related prescriptions can restrict clothing choices and public presence—thereby reducing willingness to engage in some forms of sport—religious doctrine in other contexts may actively encourage physical activity as a route to bodily health and collective wellbeing (for example, some community initiatives and policy reforms in conservative contexts have promoted women’s exercise as a form of empowerment and public health) (17, 23).

Fourth, the use of environmental resources is also subject to sociocultural intervention: university sports facilities are generally tilted toward male competitive sports, leaving women facing structural exclusion such as “outdated facilities” (FL = 0.875) and “inconvenient transportation” (FL = 0.764). The natural environment is also culturally reconstructed—ultraviolet radiation not only causes sunburn, but is also given the social metaphor of “dark skin = ugly” (10).

Therefore, in response to this multidimensional, intertwined systemic problem, we need to work together on the physiological, psychological, social, and environmental levels to empower female college students’ sports participation in practice. At the individual level, through menstrual education and injury prevention, combined with addressing confidence deficits in athletic ability, utilizing a stepwise achievement system and cognitive-behavioral restructuring of traumatic memories, we can jointly alleviate female college students’ deficiencies in physical movement cognition from both physiological knowledge and psychological intervention perspectives. At the social interpersonal network level, by establishing women-only communities, cross-cultural sports groups, and peer incentive systems, we can carve out female-dominated subcultural spaces within male-dominated sports environments. This fosters collective identity and mutual support among female college students in the process of sports participation, providing interpersonal support for their psychological barriers to sports participation. At the sociocultural level, it is necessary to deconstruct the traditional discourse system that objectifies, instrumentalizes, or marginalizes women’s bodies, and to promote female college students’ participation in sports through new narratives that emphasize agency, diverse values, and subjective experience. In terms of the participation environment, the gender politics of space production is reflected in the use of “time–space” zoning management, privacy-enhancing facility renovations, “gender protection” designs in religiously sensitive areas, and the functional transformation of idle spaces. This goes beyond the “hardware thinking” of simply adding facilities and is committed to reshaping the physical environment of sports participation into an empowering space that respects differences and guarantees safety. Crucially, these discursive changes must be paralleled by institutional commitments: universities and funding bodies should adopt transparent gender-equity criteria in sports funding allocations, integrate cultural-sensitivity requirements into facility procurement and renovation contracts, and monitor participation outcomes disaggregated by gender and cultural background to ensure accountability (8, 16).

## 6 Conclusion

Overall, the barriers to female college students’ participation in sports are the concrete reflection of gendered social structures, cultural norms, and spatial practices on individual physical experiences. In the future, we should implement systematic interventions embedded in the socio-cultural level in order to promote their sports participation. In addition, through the summary and synthesis of previous studies, this review concludes that research on female college students’ sports participation should not only discuss how to promote their participation rates, but also how to realize women’s physical practices in the sports field in the future—helping female college students to escape from their identity as “objects of gaze” in the sports field. When female college students transcend the healthy behavior represented by sports participation, it can become an important way for them to achieve physical autonomy, challenge gender norms, and participate in shaping a more equal social space. This is not only an important way to improve individual well-being, but also a key step in promoting gender equality to a deeper and more concrete realm of daily life.

In addition to this, this study acknowledges that there are still some degrees of shortcomings and flaws in the current study. Firstly, some of the influencing factors are clearly differentiated in this study (e.g., psychological and social factors), but it is important to note that there are explicit or implicit associations between these influencing factors. This study does not discuss these factors in depth, as it aims to present a narrative overview of the various influences on female university students’ participation in sport in a way that is easy for non-specialist readers to understand and accept the relevant concepts and knowledge. Future research can try to analyze the logic and correlation between these factors and present more in-depth and explanatory results. Secondly, this study is mainly a narrative review based on existing studies, and there is no longitudinal and continuous tracking or continuous intervention research on female college students, which leads to a static and isolated perspective of our research results. The changing attitudes of female college students in the process of sports participation and the dynamic challenges in reality were neglected. In addition, there are some shortcomings in the selection of research subjects in this study, which require us to pay attention to this aspect of exploration in the future.
